# A novel oncogenic BTK isoform is overexpressed in colon cancers and required for RAS-mediated transformation

**DOI:** 10.1038/onc.2015.504

**Published:** 2016-01-25

**Authors:** E Grassilli, F Pisano, A Cialdella, S Bonomo, C Missaglia, M G Cerrito, L Masiero, L Ianzano, F Giordano, V Cicirelli, R Narloch, F D’Amato, B Noli, G L Ferri, B E Leone, G Stanta, S Bonin, K Helin, R Giovannoni, M Lavitrano

**Affiliations:** 1 School of Medicine and Surgery, University of Milano-Bicocca, Monza, Italy; 2 BiOnSil srl, Monza, Italy; 3 Department of Biomedical Science, NEF-Laboratory, University of Cagliari, Monserrato, Italy; 4 Department of Medical Sciences, University of Trieste, Cattinara Hospital, Trieste, Italy; 5 Biotech Research and Innovation Centre (BRIC), University of Copenhagen, Copenhagen, Denmark; 6 Center for Epigenetics, University of Copenhagen, Copenhagen, Denmark; 7 Danish Stem Cell Center (Danstem), University of Copenhagen, Copenhagen, Denmark

**Keywords:** Colorectal cancer, Oncogenes

## Abstract

Bruton’s tyrosine kinase (BTK) is essential for B-cell proliferation/differentiation and it is generally believed that its expression and function are limited to bone marrow-derived cells. Here, we report the identification and characterization of p65BTK, a novel isoform abundantly expressed in colon carcinoma cell lines and tumour tissue samples. p65BTK protein is expressed, through heterogeneous nuclear ribonucleoprotein K (hnRNPK)-dependent and internal ribosome entry site-driven translation, from a transcript containing an alternative first exon in the 5′-untranslated region, and is post-transcriptionally regulated, via hnRNPK, by the mitogen-activated protein kinase (MAPK) pathway. p65BTK is endowed with strong transforming activity that depends on active signal-regulated protein kinases-1/2 (ERK1/2) and its inhibition abolishes RAS transforming activity. Accordingly, p65BTK overexpression in colon cancer tissues correlates with ERK1/2 activation. Moreover, p65BTK inhibition affects growth and survival of colon cancer cells. Our data reveal that BTK, via p65BTK expression, is a novel and powerful oncogene acting downstream of the RAS/MAPK pathway and suggest that its targeting may be a promising therapeutic approach.

**Supplementary information:**

The online version of this article (doi:10.1038/onc.2015.504) contains supplementary material, which is available to authorized users.

## Introduction

Bruton’s tyrosine kinase (BTK) is a nonreceptor tyrosine kinase initially identified as the defective protein in human X-linked agammaglobulinemia.^1^ Since its discovery, BTK has been considered a tissue-specific protein, being expressed throughout the hematopoietic compartment, except T cells and plasma cells. BTK plays a critical role in several hematopoietic signalling pathways including those mediated by several chemokine receptors and the B-cell antigen receptor.^2^ In B lymphocytes, as an essential component of the B-cell signalosome, BTK is involved in transducing activation, proliferation, maturation, differentiation and survival signals and is an upstream activator of multiple anti-apoptotic signalling molecules and networks, such as signal transducer and activator of transcription 5, nuclear factor-κB and the phosphatidylinositol-3-kinase/AKT/mammalian target of rapamycin pathway.^3^ BTK is overexpressed in several B-cell malignancies^3^ and different kinase-defective isoforms, exerting a dominant-negative effect over full-length BTK, have been reported in B-cell precursor leukaemia cells.^4^ Despite that its hyperactivation plays a pivotal role in chronic B-cell receptor signalling required for the survival of neoplastic B cells and that in experimental settings gain-of-function mutations providing BTK with transforming potential have been described,^2, 5, 6, 7^ no constitutively active BTK mutants have been identified so far in hematopoietic neoplasias, thus leaving the oncogenicity of BTK an open question. BTK has emerged as a new molecular target for the treatment of B-lineage leukaemias and lymphomas, and Ibrutinib is the first BTK-specific inhibitor that entered the clinic, having been recently approved for the treatment of mantle cell lymphoma and chronic lymphocytic leukaemia. Moreover, Ibrutinib and other BTK inhibitors are in advanced clinical trials for other hematological malignancies.^3^

Here, we report the identification of p65BK, a novel BTK isoform, and show that it is expressed in colon cancers and that its expression is regulated by its 5′-untranslated region (UTR) via mitogen-activated protein kinase (MAPK)/heterogeneous nuclear ribonucleoprotein K (hnRNPK)-dependent and internal ribosome entry site (IRES)-driven translation of an alternatively spliced mRNA. Moreover, we demonstrate that p65BTK is a novel and powerful oncoprotein acting downstream of the RAS/MAPK pathway and a mediator of RAS-induced transformation.

## Results

### p65BTK is widely expressed in colon carcinoma cell lines and tissues

**Figure 1:**
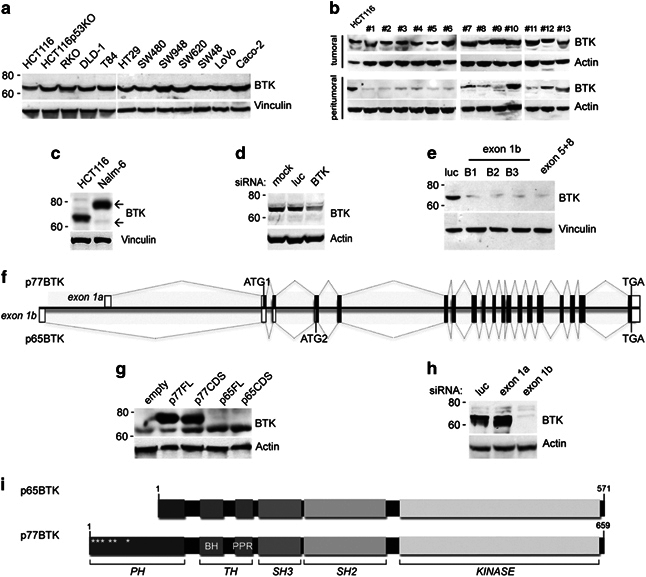
p65BTK, a novel isoform of Bruton’s tyrosine kinase, is widely expressed in colon carcinoma cell lines and tissues. (**a**, **b**) BTK expression in colon cancer cell lines (**a**) or patients’ biopsy (**b**) lysates. Western blots probed with a commercial BTK antibody (Santa Cruz, sc-1696). (**c**) Western blot showing that in colon carcinoma cells (HCT116) BTK has a lower molecular weight than in lymphoid leukaemia (Nalm-6). (**d**) Western blot of BTK expression in HCT116 cells after silencing with BTK-specific siRNA (exons 5+8). (**e**) Western blot of BTK expression in HCT116 cells upon silencing using exon 1b (B1–3)-targeting siRNAs. (**f**) *BTK* gene and mRNAs encoding p77BTK and p65BTK. ATG1 and ATG2: start codons, black/white boxes: translated/untranslated exons. Exon 1a and exon 1b are indicated. (**g**) BTK expression in 293T cells transiently transfected with empty vector (empty) and plasmids encoding p77BTK or p65BTK coding sequence (p77CDS, p65CDS), p77BTK CDS or p65BTK CDS full lengths (p77FL, p65FL). (**h**) Western blot of p65BTK expression in 293T cells transiently transfected with p65FL plasmid followed by silencing with exon1b-specific siRNAs. (**i**) p65 and p77 BTK protein organization: PH domain. BH, BTK homology region; PPR, PolyProline region; TH, Tec homology domain; *phosphoinositide binding site.

Preliminary data from our laboratory indicated that, unexpectedly, BTK is expressed in colon carcinoma cells, and thus we sought to define its function in colonic tissue. First, we observed that BTK is abundantly expressed in all colon cancer cell lines and tumour tissues analysed (Figures 1a and b). While studying the expression of BTK we noticed that its apparent molecular weight on SDS–polyacrylamide gel electrophoresis was lower than expected (Figure 1c). The downregulation of BTK expression by using specific small interfering RNA (siRNA) confirmed that the lower band is encoded by the *BTK* gene (Figure 1d). As alternative splicing of *BTK* mRNA has been reported in B-cell malignancies,^4^ we set out to identify the isoform expressed in colon cancers. Using a PCR strategy covering the entire coding sequence (CDS) of *BTK*, we were unable to amplify the 5′ of the mRNA expressed in colon cells (Supplementary Figures S1a and b). Indeed, 5′RACE (rapid amplification of cDNA ends)/sequencing experiments on colon cancer cell line-derived complementary DNAs (cDNAs) followed by ClustalW alignment (http://www.clustal.org/clustal2/) (Supplementary Figures S1c and d) revealed that colon cancer-derived mRNA contains a first exon different from the one expressed in B cells. Moreover, BLAST alignment showed that the 300 bp long exon mapped 15 192 bp upstream of the first known BTK exon (Supplementary Figure S1e). We named the exon ‘1b’, whereas the known exon 1 was referred to as ‘exon 1a’. By using isoform-specific siRNAs (Figure 1e) we confirmed that the BTK expressed in colon cancer cells is translated from exon 1b-containing mRNA and, because of its apparent molecular weight, we named it p65BTK. Analysis of p65BTK cDNA with an open reading frame (ORF) predicting program^8^ revealed—beside the expected starting codon in exon 2 (ATG1)—a putative start codon in exon 4 (Figure 1f) whose usage would lead to a predicted protein of ≈65 kDa. Transfection of 293T cells with a plasmid expressing either a putative CDS starting from the ATG in exon 4 (ATG2) or the full-length cDNA led to the expression of ≈65-kDa BTK (Figure 1g). Accordingly, siRNAs targeting exon 1b, but not those targeting exon 1a, specifically abolished the synthesis of 65 kDa isoform in overexpressing 293T cells (Figure 1h). Compared with the previously known isoforms, the predicted p65BTK protein would lack most of the N-terminal Pleckstrin homology (PH) domain (Figure 1h). To study the expression of the novel BTK isoform we then raised and characterized BN49 polyclonal antibody specific for p65BTK (Supplementary Figures S1f and g).

### hnRNPK and active ERKs post-transcriptionally regulate p65BTK expression

**Figure 2:**
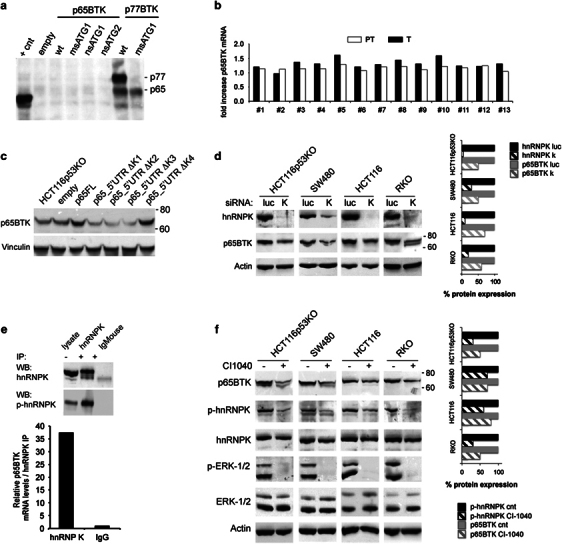
hnRNPK and active ERKs post-transcriptionally regulate p65BTK expression. (**a**) *In vitro* translation assay performed with the following plasmids: empty vector (empty); p65FL (wt), p65_msATG1, p65_nsATG1, p65_nsATG2, p77_5′UTR or p77_msATG1. +cnt indicates the positive control included in the commercial kit used for the reaction. (**b**) p65BTK mRNA expression in matched samples of tumoural and peritumoural colon tissue from CRC patients (same patients as in Figure 1b). mRNA was quantified by Taqman assay and expression levels normalized to phosphoglycerate kinase. (**c**) Western blot of 293T cells transfected with empty vector (empty) or the following plasmids: p65FL, p65_5′UTRΔK1, p65_5′UTRΔK2, p65_5′UTRΔK3, p65_5′UTRΔK4. Deletion of all four binding sites allowed p65BTK overexpression most likely by rendering the transcript as it would be a CDS. (**d**) Western blot of p65BTK levels in colon cancer cell lines after siRNA-mediated depletion of hnRNPK (K). Transfection with siRNAs targeting luciferase (luc) was used as a control. On the right, the percentage of hnRNPK and p65BTK protein expression of each sample as calculated and normalized to actin by ImageJ program (http://imagej.nih.gov/ij/). (**e**, top) Anti-hnRNPK and anti-phospho-hnRNPK western blots after RNA immunoprecipitation using anti-hnRNPK and isotype-matched control (Ig mouse) antibodies. (**e**, bottom) Real-time PCR of p65BTK mRNA recovered by RIP in hnRNPK and IgG immunoprecipitates. (**f**) Western blot of p65BTK expression and hnRNPK-Ser284 phosphorylation following ERK1/2 inhibition with the MEK1/2 inhibitor CI-1040 (10 μM). Levels of total and phospho-ERKs are also shown. Cell lysates were obtained 24 h after CI-1040 addition but for HCT116p53KO cells, where p65BTK reduction is most prominent, at 16 h. On the right, the percentage of p-hnRNPK and p65BTK protein expression of each sample was calculated and normalized to actin by ImageJ program.

To further demonstrate p65BTK production from the identified RNA we performed *in vitro* translation assays using a plasmid containing p65BTK full-length cDNA. Surprisingly, in this setting the protein was not translated, whereas small amounts of p65BTK were obtained using a plasmid bearing either wild-type p77BTK full-length cDNA or its mutated counterpart with a missense mutation in the starting codon for 77 kDa BTK (ATG1) (Figure 2a). Hence, within the context of p77BTK mRNA, the ATG2 can also be recognized as a starting codon, although with much lower efficiency.

The lack of p65BTK expression in cell-free systems, together with the observation that the high levels of protein expression in cancer tissues (Figure 1b) were not mirrored by increases of p65BTK mRNA expression in the same tissues (Figure 2b), led us to hypothesize a post-transcriptional regulation mediated by a cellular protein binding to the 5′UTR to promote the translation of exon 1b-containing mRNA. Indeed, analysis of the 5′UTR revealed the presence of four putative hnRNPK binding sites and three upstream ORFs^9^ (Supplementary Figure S2a). hnRNPK is a RNA-binding nuclear protein involved in chromatin remodelling, transcription, splicing, translation and mRNA stability,^10^ overexpressed and aberrantly localized in the cytoplasm in colorectal cancers.^11^ Indeed, transfecting p65BTK-encoding plasmids progressively deleted of the hnRNPK binding sites hampered its overexpression (Figure 2c). Moreover, p65BTK expression in colon cancer cells decreased upon silencing of hnRNPK by RNA interference (Figure 2d).

Analysis of p65BTK 5′UTR by a RNA structure prediction software (Supplementary Figure S2b) revealed a complex folding pattern, with the ATG1 hidden in a hairpin loop. We therefore hypothesized that 5′UTR-bound hnRNPK would promote a three-dimensional structure favouring the ribosome to start the translation from ATG2.

We then performed RNA immunoprecipitation (RIP) experiments to confirm the direct interaction of hnRNPK with p65BTK-encoding mRNA (Figure 2e). Previous results have shown that signal-regulated protein kinase-1/2 (ERK1/2)-mediated Ser284 phosphorylation leads to the relocalization of hnRNPK from the nucleus to the cytoplasm, where it accumulates^12^ and increases MYC mRNA translation.^13^ Interestingly, we also showed that hnRNPK (bound to p65BTK-enconding mRNA) is phosphorylated (Figure 2e), and we therefore investigated whether ERK1/2 might regulate p65BTK expression. As shown in Figure 2f, ERK1/2 inhibition (by MEK1/2 inhibitor CI-1040) indeed led to the decrease of both hnRNPK-Ser284 phosphorylation and p65BTK.

Taken together, these results demonstrate that p65BTK levels are regulated by both hnRNPK and active ERK1/2.

### hnRNPK post-transcriptionally regulates p65BTK expression via IRES-dependent translation of exon 1b-containing mRNA

**Figure 3:**
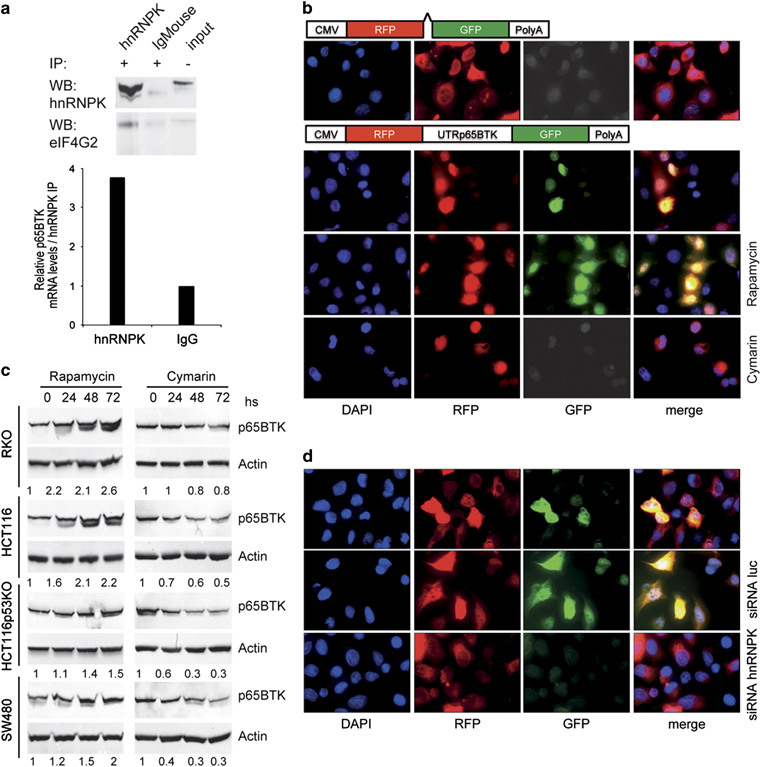
hnRNPK post-transcriptionally regulates p65BTK expression via IRES-dependent translation of exon 1b-containing mRNA. (**a**) Anti-hnRNPK antibodies immunoprecipitate a complex containing hnRNPK, eIF4G2 (top) and p65BTK mRNA (bottom) from HCT116p53KO lysates. (**b**) Fluorescence of HeLa cells transfected with a bicistronic vector encoding RFP under the control of CMV promoter and GFP not preceded by a regulatory region (first row) or under the control of p65BTK 5′UTR (second to fourth row) and left untreated (second row) or treated with Rapamycin 200 nM (third row) or Cymarin 100 nM (fourth row) for 36 h. DAPI was used to stain nuclei. (**c**) Time-dependent variation of p65BTK expression after treatment of colon cancer cells with 200 nM Rapamycin (left) and 200 nM Cymarin (right). Fold variation of p65BTK protein expression of each sample was calculated and normalized to actin by ImageJ program. (**d**) HeLa cells were transfected with the same bicistronic reporter as in (**b**) and luc-targeted siRNAs (second row) or hnRNPK-targeted siRNAs (third row). DAPI was used to stain nuclei.

The presence of several ORFs in the 5′UTR of p65BTK together with the fact that MYC translation in leukemic cells is hnRNPK dependent and IRES mediated^13^ led us to investigate whether p65BTK translation is also driven by an IRES. We identified a putative IRES in the 5′UTR of p65BTK mRNA (Supplementary Figures S3a and b) and showed that eIF4G2, a translation initiation factor involved in IRES-mediated translation,^14^ co-immunoprecipitates with hnRNPK and p65BTK-encoding mRNA (Figure 3a). Next, we verified the presence of an IRES in the 5′UTR by demonstrating green fluorescent protein (GFP) expression following transfection of HeLa cells with a bicistronic vector in which GFP translation is under the control of p65BTK 5′UTR (Figure 3b). Accordingly, GFP expression increased when the experiment was repeated in the presence of 200 nM Rapamycin—which blocks cap-dependent translation and stimulates IRES-mediated translation^15^^—^and was abolished by 200 nm Cymarin—a cardiac glycoside recently identified as a potent inhibitor of MYC IRES-mediated translation^16^ (Figure 3b). The presence of a cryptic promoter was ruled out by showing that a unique transcript coding for both red fluorescent protein (RFP) and GFP is transcribed in transfected cells (Supplementary Figure S3c). Finally, IRES-mediated translation of endogenous p65BTK was confirmed by demonstrating a time-dependent increase and decrease of p65BTK levels on treatment of colon cancer cells with Rapamycin and Cymarin, respectively (Figure 3c). Notably, in reporter assay we also demonstrated that hnRNPK is required for IRES-mediated translation of GFP, as its depletion by siRNA (Figure 3d) as well as the deletion of all hnRNPK binding sites (Supplementary Figure S4), completely abolished GFP expression.

Altogether, these data demonstrate that IRES-mediated translation of p65BTK mRNA strictly depends on hnRNPK.

### p65BTK is a novel oncogenic protein acting downstream of RAS/ERK pathway and is overexpressed in colon cancers

**Figure 4:**
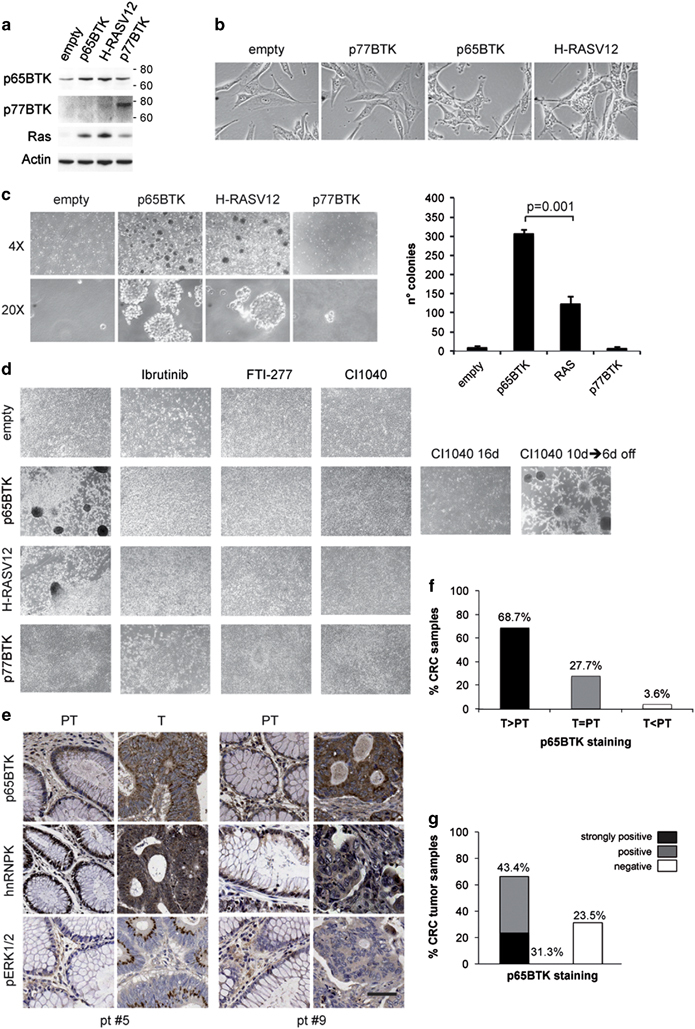
p65BTK is a novel oncogenic protein acting downstream of RAS/MAPK pathway and is overexpressed in colon cancers. (**a**) NIH3T3 cells transfected with empty vector or plasmids encoding p65BTK, p77 or mutated H-RAS (H-RASV12). p65BTK expression was assessed by p65BTK-specific polyclonal antibody BN49, whereas p77BTK was probed with a monoclonal antibody against the N-term of BTK (BD). (**b**) Phase contrast images of NIH3T3 transfected with empty vector or plasmids expressing p77BTK, p65BTK, H-RASV12; × 40 magnification. To note, p77BTK-transfected NIH3T3 maintain the same appearance of the empty vector-transfected untransformed fibroblasts, whereas p65BTK-transfected NIH3T3 are similar to H-RASV12-transformed fibroblasts. (**c**) In soft agar assay, p65BTK-transfected NIH3T3 fibroblasts showed a colony-forming activity higher than H-RASV12-transfected ones (× 10 magnification). Right: number of colonies (mean of three separate wells). (**d**) Focus assay of NIH3T3 cells transfected with empty vector, H-RASV12, p65BTK or p77BTK expression plasmids, grown in the absence or presence of BTK (Ibrutinib), RAS (FTI-277) or MEK1/2 (CI-1040) inhibitors; parallel samples of p65BTK-transfected cells were treated for 16 days with CI1040 or treated for 10 days with CI1040 followed by 6 days without drug; (× 10 magnification). (**e**) Immunohistochemical detection of p65BTK, hnRNPK and p-ERK-1/2 in formalin-fixed, paraffin-embedded specimens (× 40 magnification); tumour samples (T) showing predominant cytoplasmic hnRNPK expression and moderate to strong p-ERK-1/2 levels expressed the highest amounts of p65BTK, whereas low expression of p65BTK was detectable in peritumoural (PT) samples, in which hnRNPK was exclusively or predominantly nuclear and p-ERK-1/2 levels were very low. (**f**, **g**) Overexpression of p65BTK in patients with stage II colon cancer. Tissue microarray (TMA) analysis of p65BTK expression was performed in tumoural/peritumoural pairs of specimens from a cohort of 83 patients and results were grouped by comparing the expression in tumoural vs peritumoural tissues (**f**) and by the intensity of the staining in the tumour tissue (**g**).

In view of the abundant expression of p65BTK in colon carcinomas and its IRES-mediated translation,^17^ we suspected that p65BTK could have oncogenic properties. Indeed, transfection of a plasmid encoding full-length p65BTK (Figures 4a–d) transformed NIH3T3 fibroblasts, whereas p77BTK overexpression did not (Figure 4d). Notably, p65BTK was more potent than H-RASV12, used as a positive control, inducing more and larger colonies and foci. Inhibition of p65BTK-mediated transformation by use of the specific BTK inhibitor Ibrutinib^3, 18, 19^ indicated that p65BTK oncogenic capacity is dependent on its kinase activity. Moreover, Ibrutinib addition also blocked H-RASV12-mediated transformation (Figure 4d). Interestingly, we found that BTK overexpression in NIH3T3 cells induced high levels of endogenous RAS (Figure 4a). Even though wild-type RAS overexpression is not transforming,^20, 21, 22, 23^ its expression appeared necessary for p65BTK-mediated transformation, as the RAS inhibitor FTI277, as well as cotransfection with a RAS-DN plasmid, abolished p65BTK-mediated transformation of NIH3T3 cells (Figure 4d and Supplementary Figure S5b). Conversely, H-RASV12 overexpression increased endogenous p65BTK (Figure 4a) and endogenous RAS knockdown rapidly depleted p65BTK (Supplementary Figure S5a), confirming that RAS indeed regulates p65BTK expression. However, p65BTK silencing did not affect endogenous RAS expression (Supplementary Figure S5a), suggesting that the observed endogenous RAS induction in p65BTK-transfected NIH3T3 cells is an effect of exogenous p65BTK overexpression. Finally, p65BTK-mediated transformation was suppressed when blocking RAS/MAPK pathway downstream of RAS, namely by using MEK1/2-inhibitor CI-1040 (Figure 4d). Altogether, these data indicate that p65BTK is an obligate effector of activated RAS.

We then confirmed our results showing that p65BTK expression parallels ERK1/2 activation and abnormal hnRNPK cytoplasmic localization by immunohistochemical analysis on paired peritumoural/tumoural samples from the same 13 colon carcinoma patients whose tissues have already been analysed for p65BTK expression in Figures 1b and 2b (Figure 4e, Supplementary Figure S6 and Supplementary Table S1).

Furthermore, we analysed p65BTK expression in a cohort of 83 stage II colon carcinoma patients and found that in 68.7% of peritumoural/tumoural sample pairs, p65BTK was more expressed in tumoural than in peritumoural tissue (Figure 4f); in addition, the grading of p65BTK according to an increasing intensity of the staining in tumoural samples (Supplementary Figure S7) showed moderate to high levels of the protein in the 74.7% of colon cancer tissues analysed (Figure 4g).

Taken together, our results suggest that p65BTK is an oncoprotein whose expression and transforming activity are tightly controlled, via hnRNPK, by the RAS/ERK pathway and that p65BTK overexpression in colon carcinomas reflects hyperactivation of the RAS/ERK pathway.

### p65BTK inhibition affects growth and survival of colon cancer cells

**Figure 5:**
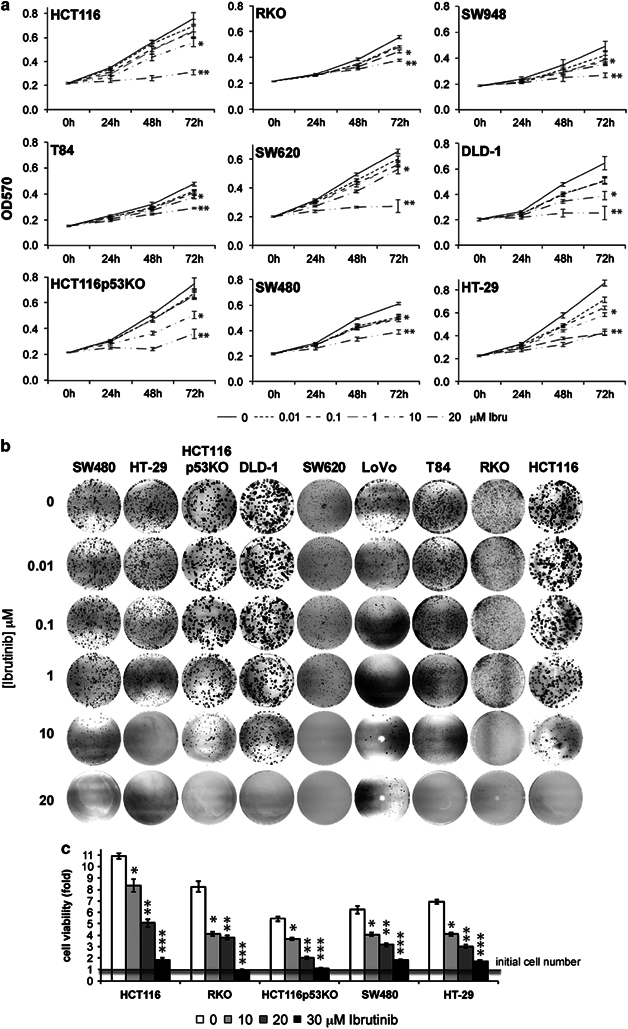
p65BTK inhibition affects growth and survival of colon cancer cells. (**a**) Time course showing Ibrutinib dose response (0, 0.01, 0.1, 1, 10, 20 μM Ibru) of colon carcinoma cell lines characterized by different genetic background; cell proliferation was determined every 24 h by MTT assay on cells incubated with Ibrutinib at the indicated concentrations; error bars show s.e.m.; data are the average of 3–5 independent experiments. Ibrutinib at 10 and 20 **μ** M significatively decreases cell growth in all cell lines *10 vs 0 **μ** M Ibru *P*<0.05; **20 vs 0 **μ** M Ibru: *P*<0.05. (**b**) Clonogenicity was assessed by seeding cells at low density and incubating them with the indicated doses of Ibrutinib for 10–12 days, at the end of which colonies were stained by crystal violet. (**c**) Cell viability was assessed after 72 h of treatment with the indicated concentration of Ibrutinib; crystal violet assay was performed to quantify viable cells; data are presented as fold change of the initial cell number obtained from 3 independent experiments; error bars show s.e.m. *10 vs 0 **μ** M Ibru: *P*<0.05; **20 vs 0 **μ** M Ibru: *P*<0.05; ***30 vs 0 **μ** M Ibru: *P*< 0.05.

Finally, we tested the requirement of p65BTK in colon cancer cell biology. For all colon cancer cell lines tested, *in vitro* dose–response experiments showed that concentrations up to 10 μM Ibrutinib caused a slight to moderate decrease in proliferation in the short term (Figure 5a) and strongly affected clonogenicity in the long term (Figure 5b); higher doses further inhibited the proliferation of all cell lines and completely suppressed cell growth at 30 μM (Figures 5a and c) concomitantly with a significant increase of cell death (Supplementary Figure S8a). Similar results were obtained treating colon cancer cell lines with AVL-292, a different BTK inhibitor also in clinical trials for treating B-cell malignancies.^19^ Notably, AVL-292 at 10 μM almost completely suppressed cell growth and had a mild but significant cytotoxic effect (Supplementary Figure S8b) that increased in a dose-dependent manner (Supplementary Figure S8c).

## Discussion

Since its discovery, BTK has been considered a tissue-specific kinase expressed only in bone marrow-derived cells.^2^ In particular, BTK transduces essential signals for the proliferation and differentiation of B lymphocytes and it has been found overexpressed/constitutively active in several B-lineage lymphoid malignancies.^3^ Here we report the identification and characterization of p65BTK, a novel oncogenic isoform, whose 5′UTR-regulated expression is finely tuned downstream of ERK1/2 activation via hnRNPK- and IRES-dependent translation, whose activity is required for H-RASV12-induced transformation and whose levels are increased in a high percentage of colon cancers.

The most striking finding of this paper is that not only is BTK expressed outside of the hematopoietic compartment but, via p65BTK expression, is also a potent oncogene. Different kinase-defective isoforms of BTK have been reported in B-cell precursor leukaemia cells,^4^ and an 80-kDa isoform, bearing an extended N-term domain, has been demonstrated in breast carcinoma cells^24^ and at least three other protein-coding splice variants can be predicted by the Ensembl automatic gene annotation system (http://www.ensembl.org/Homo_sapiens/Transcript/Summary?db=core;g=ENSG00000010671;r=X:101349447-101386224;t=ENST00000621635). However, this is the first time that the expression of an isoform lacking most of the PH domain is found (Figure 1). By binding phosphatidylinositol-3-kinase-generated phosphatidylinositol-3,4,5-trisphosphate, PH domain allows BTK translocation to the plasma membrane and its activation.^2^ Several other proteins have been reported to interact with BTK via the PH domain, most of them negative regulators: protein kinase C-β binding interferes with plasma membrane targeting and subsequent activation of BTK;^25, 26^ inhibitor of BTK physically associates with BTK and downregulates its kinase activity;^27, 28^ the peptidyl-prolyl cis-trans isomerase Pin1, by binding to S21 and S115, leads to the destabilization of the protein.^29^ It is therefore likely that because of the absence of most of the PH domain, p65BTK would be regulated/activated differently than p77BTK, as well as be involved in different signalling pathways. Moreover, lacking the region responsible for its negative regulation, it may be expected that p65BTK would be abundantly expressed and activated. Indeed, at variance with p77BTK, p65BTK is endowed with a strong transforming activity (Figure 4). The transforming potential of BTK has been matter of debate since its discovery and has never been completely resolved. It has been demonstrated that gain-of-function mutations introduced experimentally in the PH domain provide BTK with transforming potential;^2, 5, 6, 7^ however, no constitutively active BTK mutants have been identified so far in hematopoietic neoplasias, although it has been extensively shown that p77 plays pro-survival and anti-apoptotic roles in B cells.^2, 3^ Recently, a 80-kDa isoform, bearing an extended N-term, has been identified by Eifert *et al.*^24^ in breast carcinoma cells having, similar to p77BTK, pro-survival and anti-apoptotic roles. As for the transforming potential of BTK, our results clearly indicate that overexpression of p77BTK is not transforming, whereas overexpression of p65BTK is even more powerful than H-RASV12 in transforming NIH-3T3 cells (Figures 4c and d). We therefore conclude that BTK is indeed an oncogene, being its transforming activity carried out by the p65, but not the p77, isoform.

**Figure 6:**
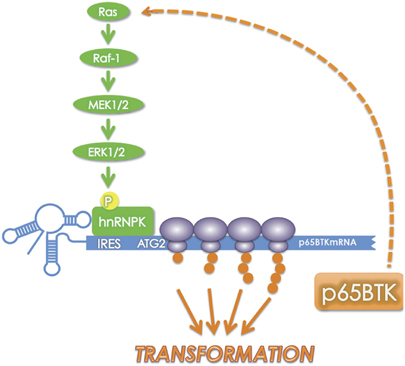
Proposed model of p65BTK regulation.

A main point of the paper is that p65BTK expression and oncogenicity result from RAS/ERK pathway activation (Figure 6). Several lines of evidence demonstrate that p65BTK (over)expression is controlled, via hnRNPK, by the RAS/ERK pathway. p65BTK mRNA-bound hnRNPK is phoshorylated on Ser284 (Figure 2e), a residue known to be phosphorylated by ERK1/2.^12, 13^ Accordingly, upon blocking ERK1/2 activation p65BTK levels decreased concomitantly to hnRNPK-p-Ser284 reduction (Figure 2f). Notably, ERK1/2-mediated Ser284 phosphorylation leads to the relocalization of hnRNPK from the nucleus to the cytoplasm^12^ and a cytoplasmic localization is necessary for hnRNPK to participate in p65BTK mRNA translation. In addition, p65BTK-mediated transformation is suppressed in the presence of CI-1040 but resumes when the inhibitor is removed from the medium (Figure 4d), consistent with a restart of ERK/hnRNPK-mediated translation of p65BTK mRNA. Accordingly, also blocking the RAS/ERK pathway upstream of ERK1/2, that is, by inhibiting endogenous RAS either by use of a chemical inhibitor or a RAS-DN, abolished p65BTK-mediated transformation of NIH-3T3 cells (Figure 4d and Supplementary Figure 5b). Even though it has been demonstrated that overexpression of wild-type RAS, at variance with mutated RAS, does not transform NIH3T3 cells,^20, 21, 22, 23^ a p65BTK-mediated increase in endogenous RAS levels may enhance p65BTK transforming activity by triggering a positive feedback loop. A possibility might be that p65BTK directly, or via one or more effector(s), induces RAS expression or blocks its degradation: such a mechanism would justify the stronger transforming activity of p65BTK compared with H-RASV12. Additional studies are required to ascertain this hypothesis. Conversely, p65BTK inhibition (Figure 4d) also prevented H-RASV12-mediated transformation, indicating that p65BTK is a pivotal downstream effector of RAS and confirming that its transforming activity depends on the RAS/ERK pathway. Finally, we showed in paired peritumoural/tumoural samples from colon carcinoma patients that p65BTK expression parallels ERK1/2 activation and abnormal hnRNPK cytoplasmic localization (Figure 4e). A further indication that p65BTK is key effector in the RAS/ERK pathway is given by the results obtained on its inhibition in colon cancer cells. It is well known that the RAS/ERK pathway is critical for transducing mitogenic signals and regulating cell proliferation.^30^ Accordingly, p65BTK inhibition profoundly affects proliferation and clonogenicity of all colon cancer cells tested (Figure 5). Given that deregulation of the RAS/ERK pathway^31^ occurs at high frequency in colon cancers, our data indicate that p65BTK might be a novel promising therapeutic target in this kind of tumours.

A comprehensive analysis of the mammalian transcriptome showed that most genes allow the expression of alternative 5′UTRs resulting either by use of multiple transcriptional start sites or by differential splicing.^32^ Alternative 5′UTRs may allow transcript isoforms to bind different RNA-binding proteins, thus leading to tissue-specific or stage-specific expression.^33^ Moreover, inappropriate expression of alternative 5′UTRs can contribute to tumourigenesis as in case of alternative 5′UTRs regulating the translation of BRCA1, MDM2 and transforming growth factor-β.^32^ All the examples reported so far in the literature show that tissue-specific, stage-specific or inappropriate expression of transcripts bearing alternative 5′UTRs control the expression of the same CDS, making it subject to developmental, physiological or pathological regulation. Our results demonstrate for the first time that alternative 5′UTRs can contribute to the diversification of gene expression by also driving the production of different protein isoforms, endowed with different functions.

Several oncogenic proteins can be translated by both cap-dependent and IRES-dependent mechanisms, the latter being switched on to maintain the expression of specific proteins during pathological situations when cap-dependent translation is compromised.^34^ Interestingly, p65BTK translation is strictly IRES dependent (Figures 3b and c), suggesting that its expression should be very low in physiological conditions or in nontransformed cells. Moreover, in the 5′UTR of p65BTK mRNA, three upstream ORFs are present (Supplementary Figure S2a) that, in unstressed conditions, reduce the efficiency of translation initiation of the main downstream ORF.^35^ Indeed, basal levels of p65BTK are low in immortalized NIH-3T3 cells (Figure 4a) and very low or undetectable in peritumoural samples (Figures 1b and 4e and Supplementary Figure S6). Translational control is a crucial component of cancer development and progression, and a role for RAS/ERK signalling pathway in the regulation of cap-dependent translation via its action on mammalian target of rapamycin complex 1 is well accepted.^36^ Our data about ERK/hnRNPK-dependent regulation of IRES-driven translation of p65BTK, together with the demonstration that RAS-induced transformation requires p65BTK, suggest that RAS/ERK signalling, via hnRNPK, may also play a crucial role in the regulation of IRES-dependent translation and that disregulation of IRES-mediated translation may be a feature of cancer cells with an hyperactive RAS/ERK pathway (like colon cancer cells).

In conclusion, we show that a novel isoform of BTK is expressed outside of the hematopoietic compartment as a result of a complex post-transcriptional mechanism, and we provide evidence that alternative 5′UTRs can contribute to the diversification of gene expression by driving the production of different protein isoforms, endowed with different transforming potential. Moreover, our results demonstrating that BTK is a potent oncoprotein acting downstream of the RAS/ERK pathway, together with those showing that its inhibition profoundly affects colon cancer cells proliferation and survival, suggest that p65BTK might be a novel promising therapeutic target in colon cancer, where deregulation of the RAS/ERK pathway occurs at a very high frequency.

## Materials and methods

### Plasmids

Standard cloning methods were used to generate all plasmids, whereas 5′ RACE was performed to clone 5′ end of p65BTK mRNA. Detailed methods are described in the Supplementary Methods.

### Cell lines, culture and treatments

Isogenic p53wt (HCT116) and p53KO (HCT116p53KO) HCT116 colon carcinoma cell lines were from Dr Bert Vogelstein (Johns Hopkins University, Baltimore, MD, USA) through the GRCF Biorepository & Cell Center of the John Hopkins School of Medicine. The 293T, HeLa, DLD-1, SW480, RKO, T84, HT-29, SW948, SW620, SW48, LoVo, CaCo-2 and NIH3T3 and HeLa cells were from American Type Culture Collection (LGC Standards, Sesto San Giovanni, Italy). Nalm-6 were from Deutsche Sammlung von Mikroorganismen und Zellkulturen GmbH (Braunschweig, Germany). All the repositories guaranteed cell line identity by genotypic and phenotypic testing. Upon arrival, cells were expanded and frozen as seed stocks of first or second passage. All cells were passaged for a maximum of 6 weeks, after which new seed stocks were thawed for experimental use. All cells were grown at 37 °C in 5% CO_2_ and were maintained as a subconfluent monolayer in McCoy medium (HCT116, HCT116p53KO, DLD-1, SW480, HT-29, SW620), Dulbecco’s modified Eagle’s medium/Ham’s F12 (T84), RPMI-1640 (Nalm-6, SW48, SW948), Ham’s F12 (LoVo) or Dulbecco’s modified Eagle’s medium (NIH3T3, 293T, HeLa, RKO, Caco-2) supplemented with 10% fetal bovine serum (except for NIH3T3 cells medium, supplemented with 10% calf serum) and 1% penicillin/streptomycin; 1% nonessential amino acids was also added to RKO and Caco-2 medium. Cells were routinely checked for mycoplasma contamination each time a new stock was thawed. Media, serum and supplements were all from Invitrogen (Life Technologies Italia, Monza, Italy) except for calf serum (Colorado Serum Company, Denver, CO, USA). Ibrutinib and AVL-292 (Selleckchem, Houston, TX, USA) were dissolved in dimethyl sulfoxide and stored in aliquots at −80 °C.

### Transfection and silencing experiments

The siRNA and plasmid transfections were performed using Lipofectamine 2000 (Invitrogen) according to the manufacturer’s instructions. Silencing experiments and siRNA sequences are described in detail in Supplementary Information. Each transfection and silencing experiment was repeated at least three times.

### Cell transformation assays

#### Focus assay

NIH3T3 cells were seeded at 70% confluency in a 6-well plate the day before and then were transfected using Lipofectamine 2000 and 4 μg DNA/well; 36 h after transfection, cells were reseeded in triplicate in 6-well plate in the presence or absence of inhibitors of BTK (Ibrutinib, 10 μM), RAS (FTI-277, 10 μM) and MEK1/2 (CI-1040 10 μM). Inhibitors were replenished each day, whereas medium was changed every other day. After 10 days, foci were fixed and stained in 1% crystal violet, 35% ethanol. Parallel samples of p65BTK-transfected cells were treated for 16 days with CI1040 or treated for 10 days with CI1040 followed by 6 days without drug.

#### Soft agar assay

An aliquot (1000 cells) of NIH3T3 cells transfected as above were resuspended in warm (37 °C) 0.4% Top Agar Solution and seeded on a solidified 0.8% Base Agar Solution, both prepared according to the protocol of the Cell Transformation Detection Assay (Merck-Millipore, Vimodrone, Italy). Cells were fed every 3 days with cell culture medium and colonies counted after 10 days by 3 independent evaluators. Cell transformation assays were repeated three times.

### Cell growth/proliferation assay

5 × 10^3^ cells per 96-well plate were seeded in triplicate, and starting the following day (day 0) proliferation was evaluated every 24 h using a MTT-based assay (Sigma-Aldrich, Milano, Italy) according to the manufacturer’s instructions. Graphs represent the average of three to five independent experiments. Average±s.e.m. are plotted in the graphs.

### Colony assay

Cells were seeded at low density (1000 cells/well in 6-well plate) in triplicate and left untreated or treated with different concentrations of Ibrutinib. Medium (alone or contanining Ibrutinib) was replaced every other day, and after 10 days colonies were fixed and stained in 1% crystal violet, 35% ethanol. Colony assays were repeated three times.

### Cell viability

Cells were seeded in sextuplicates at 70% confluency the night before and the next morning treated or not with the indicated concentrations of Ibrutinib. After 72 h, cell viability was evaluated by crystal violet staining. Briefly, after washing with phosphate-buffered saline, cells were fixed/stained with a solution of 0.5% crystal violet in 20% methanol for 20 min at room temperature and then washed extensively with tap water. Colour was extracted by adding 0.1 M acetic acid and quantified by spectrofotometer at 595 nm. Graphs represent the average of three separate experiments. Average±s.e.m. are plotted in the graphs.

### GFP/RFP fluorescence assay

Cells transfected with GFP/RFP bicistronic vectors were harvested after 36 h, fixed with 4% paraformaldehyde in phosphate-buffered saline and counterstained with DAPI (4′,6-diamidino-2-phenylindole). Fluorescence microscope examination was performed using a Nikon Eclipse 80i microscope at × 60 magnification. Images were acquired using Genikon (Nikon Instruments, Campi Bisenzio, Italy) software and processed with Adobe Photoshop. GFP/RFP fluorescence assays were repeated three times.

### Tissue samples

Permission for using tissue specimens surgically removed from patients was granted by the ethical committee of the University of Milano-Bicocca. Multiple specimens, collected from patient admitted to Desio Hospital (*n*=13, for patient characteristics see Supplementary Table S1), were dissected by a pathologist from matched peritumoural/normal tissues removed during surgery and either immediately frozen at −80 °C for RNA and protein analysis or routinely fixed in formalin for subsequent hystological and immunohistochemistry analysis on tissue microarray. Frozen specimens were used to measure p65BTK expression by quantitative PCR after processing with RNeasy kit (Qiagen, Milano, Italy) and by western blot upon tissue lysis in RIPA buffer, as described below. In a separate analysis, tissue microarray samples from a cohort composed of 83 patients (admitted to Trieste University Hospital; for patients characteristics see Supplementary Table S1) with a clinical diagnosis of colon cancer, classified by a pathologist as stage II, were examined for p65BTK expression by immunohistochemistry.

### Immunohistochemistry

Specimens from patients admitted to Desio Hospital (*n*=13) were fixed with formalin, dehydrated, diaphanized with xylene, put in paraffin and processed for tissue microarray. Slides were stained according to standard immunohistochemistry procedures with the following primary antibodies: anti-hnRNPK (sc-25373) from Santa Cruz Biotechnologies (Heidelberg, Germany); phospho-ERK (Thr202/Tyr204) (#4370) from Cell Signaling (Danvers, MA, USA); and anti-p65BTK BN49 polyclonal antibody. Slides were digitally acquired using Aperio ScanScope System (Leica Microsystems, Milano, Italy). On specimens from patients admitted to Trieste University Hospital (*n*=83), p65BTK staining was graded accordingly to an increasing intensity by blind reading by two experienced operators and classified as negative, positive and strongly positive.

### RNA extraction and RIP

RNA was isolated using an RNeasy kit (Qiagen) following the manufacturer’s instructions. In RIP experiments, RNA was purified from anti-hnRNPK (ab39975, Abcam, Cambridge, UK) immunoprecipitated complex from colon cancer cell (HCT116p53KO) lysates using the Magna RIP kit (Millipore, Vimodrone, Milano, Italy) following the manufacturer’s instructions. Isotype matched antibodies were used as a control. RIP experiments were repeated three times.

### PCR

End point PCR, 5′RACE PCR and real-time PCR procedures and primers are described in Supplementary Information.

### Anti-p65BTK antibody production and characterization

BN49 polyclonal antibody produced by immunizing rabbits with a GST fusion protein encompassing the first 30N-term aa of p65BTK absorbed to nanogold.^37^ Antisera specificity was assessed by western blot analysis on lysates from p65BTK-expressing and p65BTK-silenced cells (Supplementary Figure 1f) and used in all western blots to probe p65BTK unless differently specified. In immunocytochemistry, specificity was additionally tested using pre-immune serum, as well as by pre-absorption with corresponding synthetic peptide/s (up to ∼50 nmol/ml) on sections from cell blocks of SW480 p65BTK-expressing and p65BTK-silenced cells (Supplementary Figure 1g) and on sections from colon cancer patient tissues.

### Western blot analysis

Protein extracts were prepared using high-salt lysis buffer (Hepes 50 mM, pH 7.5, NaCl 500 mM, DTT 1 mM, EDTA 1 mM, 0.1% NP-40) supplemented with 1% protease inhibitor cocktail (Sigma-Aldrich). Then, 10–20 μg cell and tissues lysates were separated on 10% NuPAGE gels (Invitrogen), transferred onto a nitrocellulose membrane (Invitrogen) and incubated with the following antibodies: anti-p65BTK (BN49); anti-BTK (sc-1696) anti-hnRNPK (sc-25373) from Santa Cruz Biotechnologies; anti-ERK (#9101), anti-phospho-ERK (Thr202/Tyr204) (#4370), anti-eIF4G2 (#5169) from Cell Signaling; anti-actin (A1978), anti-vinculin (V9264), anti-phospho-hnRNPK (SAB4504229) from Sigma-Aldrich; and anti-RAS (#05-516) from Millipore. Each single blot was reprobed with anti-actin or anti-vinculin as loading control. Images were acquired using G:BOX XT4 Chemiluminescence and Fluorescence Imaging System (Syngene, Cambridge, UK) and processed with Adobe Photoshop.

### *In vitro* translation

TnT Quick Coupled Transcription/Translation Systems (Promega, Milano, Italy) has been used according to the manufacturer’s instructions. Briefly, 1 μg each plasmid DNA was mixed with 12.5 μl Master mix from the kit and 1 μl Transcend Biotinylated tRNA (Promega). Translated products, separated on NuPAGE and blotted onto nitrocellulose, were detected by chemiluminescence upon incubation with the horseradish peroxidase/streptavidine conjugate. The *in vitro* translation experiments were repeated three times.

### Statistical analysis

The *t*-test was applied to evaluate statistically significant differences between series of samples subjected to different experimental treatments, and *P⩽*0.05 was considered significant.

## Supplementary information

Supplementary Information (PDF 2631 kb)

Supplementary Table 1 (PDF 46 kb)
