# Correction: A novel oncogenic BTK isoform is overexpressed in colon cancers and required for RAS-mediated transformation

**DOI:** 10.1038/s41388-024-03037-w

**Published:** 2024-04-18

**Authors:** E. Grassilli, F. Pisano, A. Cialdella, S. Bonomo, C. Missaglia, M. G. Cerrito, L. Masiero, L. Ianzano, F. Giordano, V. Cicirelli, R. Narloch, F. D’Amato, B. Noli, G. L. Ferri, B. E. Leone, G. Stanta, S. Bonin, K. Helin, R. Giovannoni, M. Lavitrano

**Affiliations:** 1 School of Medicine and Surgery, University of Milano-Bicocca, Monza, Italy; 2 BiOnSil srl, Monza, Italy; 3 NEF-Laboratory, Department of Biomedical Science, University of Cagliari, Monserrato, Italy; 4 Department of Medical Sciences, University of Trieste, Cattinara Hospital, Trieste, Italy; 5 Biotech Research and Innovation Centre (BRIC), University of Copenhagen, Copenhagen, Denmark; 6 Center for Epigenetics, University of Copenhagen, Copenhagen, Denmark; 7 Danish Stem Cell Center (Danstem), University of Copenhagen, Copenhagen, Denmark

Correction to: *Oncogene* 10.1038/onc.2015.504, published online 25 January 2016

Following the publication of this article, an error was detected in Figure 1b. Upon re-checking data, it was noted that the incorrect blot relating to the expression of actin in the peritumoral tissue of patients 11, 12, 13 was published. The corrected Figure 1b is provided below.
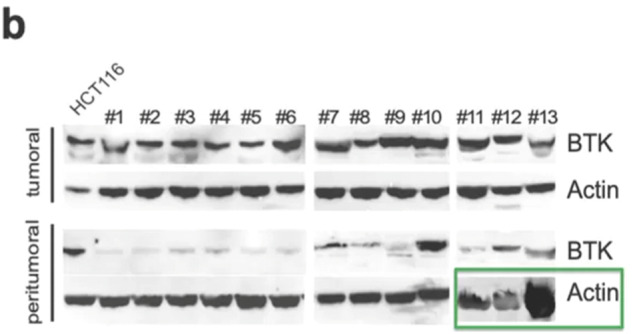


The authors confirm the results and conclusions of this paper remain unchanged and apologise for any inconvenience caused.

