# Supplementary material for: A novel oncogenic BTK isoform is overexpressed in colon cancers and required for RAS-mediated transformation
Source: Oncogene. 2016 Jan 25;35(33):4368–78. doi: 10.1038/onc.2015.504 (PMC4994017; doi:10.1038/onc.2015.504)
Supplement: Supplementary file 1 — Supplementary Information (PDF 2631 kb) [file 41388_2016_BFonc2015504_MOESM54_ESM.pdf]

SUPPLEMENTARY INFORMATIONS

SUPPLEMENTARY METHODS

Plasmids

Full length p65BTK was amplified from HCT116p53KO-derived cDNA using primers #1 and #2 (see below for primers' sequences) and cloned into pGEM vector (Promega) to originate pGEM-FLp65. p65FL, the p65BTK-expressing vector was created by PCR subcloning the entire p65BTK sequence from the pGEM-FLp65 plasmid into pcDNA3.1 (Invitrogen) using primers #3 and #4. p65CDS was created by PCR subcloning the p65BTK CDS from pGEM-FLp65 plasmid with primers #5 and #6, designed to exclude the 5' and 3'UTRs. p65_5'UTR**Δ**k1, p65_5'UTR**Δ**k2, p65_5'UTR**Δ**k3, p65_5'UTR**Δ**k4 were created by PCR subcloning into pcDNA3.1 p65BTK amplified from pGEM-FLp65 using reverse primer #4 and as forward primers oligonucleotides annealing downstream of the first (primer #7), second (primer #8), third (primer #9) and fourth (primer #10) hnRNPK binding site, respectively. p65BTK_msATG1, _nsATG1 and _nsATG2 were created via site-directed mutagenesis (QuikChange Site-Directed Mutagenesis Kit, Stratagene), according to manufacturer instructions'. As a template p65FL plasmid was used together with primers #11 and #12 to introduce a missense (ms) or with primers #13 and #14 to introduce a non-sense (ns) mutation into the ATG1, or with primers #15 and #16 to introduce a ns mutation into the ATG2. Full length p77BTK was amplified from Nalm6-derived cDNA using primers #1 and #17 and cloned into pGEM vector to originate pGEM-FLp77. p77FL was created by PCR subcloning the entire p77BTK sequence from the pGEM-FLp77 plasmid into pcDNA3.1 using primers #18 and #4. p77CDS was created by PCR subcloning p77BTK from pGEM-FLp77 plasmid into pcDNA3.1 with primers #19 and #6, designed to exclude the 5' and 3'UTRs. p77BTK_msATG1 was created by introducing via site-directed mutagenesis a ms mutation into the ATG1 of p77BTK from the p77FL plasmid using primers used #10 and #11. To create the bi-cistronic vectors, Red Fluorescent Protein (RFP) and Green Fluorescent Protein (GFP) were amplified from

pDsRed2-N1 (Clontech) (primers #21 and #22)and pcDNA3.1-EGFP (Invitrogen) (primers #23 and #24), respectively, then independently subcloned into pGEM vector (pGEM-RFP and pGEM-GFP).The whole 5'UTR of p65BTK and **Δ**k1/**Δ**k2/**Δ**k3/**Δ**k4-deleted counterparts were amplified from pGEM-FLp65 (reverse primer #4 and forward primers #3, #7, #8, #9 and #10 respectively) and ligated upstream of GFP in pGEM-GFP: the resulting products were first subcloned downstream RFP in pGEM-RFP (*EcoR*I/*Xba*I excision), and successively the whole sequence RFP-5'UTR (**Δ**k1/**Δ**k2/**Δ**k3/**Δ**k4)-GFP was cut out (*BamH*I/*Xba*I digestion) and cloned into pcDNA3.1 to create RFP-UTRp65BTK-GFP, RFP-UTRp65**Δ**k1-GFP, RFP-UTRp65**Δ**k2-GFP, RFP-UTRp65**Δ**k3-GFP, RFP-UTRp65**Δ**k4-GFP, respectively. pSuper plasmids targeting BTK and H-RAS were made by inserting a 19-bp target sequence (BTK: 5'-TTTCTATGGAGTCTTCTGC-3'; H-RAS: 5'-GGCAAGAGTGCGCTGACCATC-3') in pSuper plasmid (Oligoengine) cloned in both sense and antisense orientations, separated by a loop sequence, in order to obtain the transcription of a short-hairpin RNA. pcDNA3-RAS-DN was made by subcloning RAS-DN in pcDNApcDNA3. 3-HRASV12 was from K. Helin lab. pRSV-RAS-DN and pSuperior-shRAS were kind gifts of Dr. Elena Sacco (University of Milano-Bicocca) and Dr. Luca Mologni (University of Milano-Bicocca), respectively.

#1: 5' GTAATTTTATTTTATCAAAACACCCTC 3'

#2: 5' TCTTTTGGTGGACTCTGCTACG 3'

#3: 5' GGATCCTCTTTTGGTGGACTCTGCTACG 3'

#4: 5' GTAATTTTATTTTATCAAAACACCCTC 3'

#5: 5' CTCGAGAAATGGAGCAAATTTCAATCAT 3'

#6: 5' GGGCCCGGCGAGCTCAGGATTCTTCA 3'

#7: 5' GGATCCCAGAAAAAGAAAACATCACCTCTA 3'

#8: 5' GGATCCGAGGGAAGCCAGGACTAGG 3'

#9: 5' GGATCCCGTCCCCGAGGGAAGC 3'

#10: 5' GGATCCAGATCCTCTGGCCTCCCC 3'

#11: 5’GTGAACTCCAGAAAGAAGAAGCTTTGGCCGCAGTGATTCTG 3’

#12: 5’CAGAATCACTGCGGCCAAAGCTTCTTCTTTCTGGAGTTCAC 3’

#13: 5’ GTGAACTCCAGAAAGAAGAAGCTTAGGCCGCAGTGATTCTG 3’

#14: 5’CAATGATTGAAATTTGCTCCTATCACTGGACTCTTCACCTC 3’

#15: 5’GAGGTGAAGAGTCCAGTGAATAGAGCAAATTTCAATCATTG 3’

#16: 5’CAATGATTGAAATTTGCTCTATTCACTGGACTCTTCACCTC 3’

#17: 5’ AACTGAGTGGCTGTGAAAGG 3’

#18: 5' GGATCCAACTGAGTGGCTGTGAAAGG 3’

#19: 5' CTCGAGTCATGGCCGCAGTGATTCTG 3'

#20: 5' GCGGCCGCTTCACTGGACTCTTCACCTCT 3'

#21: 5' GGATCCACCATGGCCTCCTCCG 3'

#22: 5' GAATTCTTAAGATCTCAGGAACAGGTGG 3'

#23: 5' GCGGCCGCCTAGAATGGCTAGCAAAGGA 3'

#24: 5' TCTAGATTATTTGTAGAGCTCATCCATGC 3'

Silencing experiments. For silencing endogenous BTK in colon cancer cell lines a mix of siRNA targeting sequences in exon 5 (nts 895-913) and exon 8 (nts: 518-536) were used. For silencing specifically p65BTK, exon 1b was targeted by using each of the following siRNAs: B1 (nts: 160-182), B2 (nts: 237-259), B3(ntss: 179-197). For silencing specifically p77BTK, exon 1a was targeted by using siRNA for a sequence between nts 78-100. For silencing exogenous overexpressed p65BTK in 293T cells, the combination of B1, B2 and B3 siRNAs was used. For silencing hnRNPK in colon cancer cell lines a mix of 3 different siRNA (s6737, s6738, s6739 Silencer® Select from Invitrogen) was used. Luciferase siRNAs (Luciferase GL2) were from Eurofins MWG Operon. In all silencing experiments cells were harvested and lysed 48 hs after the silencing, protein extracts were separated on 10% NuPAGE gels and western blotted as described below.

exon 5: 5’GGGAAAGAAGGAGGTTTCA 3’

exon 8: 5’GAAGCTTAAAACCTGGGAG 3’

B1: 5’GAACACCTTTCGCAGCAAACTG 3’

B2: 5’ GCCAGTTGGTCCATTCAACAAAT 3’

B3: 5’ ACTGCTAATTCAATGAAGA 3’

exon 1a: 5’CAGTGTCTGCTGCGATCGAGTCC 3’

PCR. *Endpoint PCR.* Purified RNA was retrotranscribed using SuperScript VILO cDNA Synthesis kit (Invitrogen). To identify the BTK isoform expressed in colon cancer cell lines amplification was performed using 200 ng cDNA and 0.4 μM of the following primers, covering the entire coding sequence (CDS) of BTK (NM_000062). cDNA from NALM-6 was used a a control.

1F: 5’ CTCAGA CTGTCCTTCCTCTC 3’

8R: 5’GTTGCTTTCCTCCAAGATAAAAT 3’

2F: 5’ATCCCAACAGAAAAAGAAAACAT 3’

8F: 5’ATCTTGAAAAAGCCACTACCG 3’

14F: 5’CTCAAATATCCAGTGTCTCAACA 3’

12F: 5’TGATACGTCATTATGTTGTGTGTT 3’

14R: 5’ATCATGACTTTGGCTTCTTCAAT 3’

17R: 5’CTTTAACAACTCCTTGATCGTTT 3’

19R: 5’TCAGGATTCTTCATCCATGACATCTA 3’

HeLa cDNA was amplified as above using the following primers, targeting the RPF/GFP sequence:

RED-F : 5' GACATCCCCGACTACAAGAAG 3'

GFP-R: 5' GAAAGGGCAGATTGTGTCG 3'

PCR products were separated on 1% gel and visualized upon ethidium bromide staining.

5'RACE. Amplification of 5' end of p65BTK mRNA was performed using the 5' RACE System for Rapid Amplification of cDNA Ends from Invitrogen, following manufacturer's instructions. For 5' end identification RNA extracted from colon cancer cell lines was used. PCR products were ligated in pGEM-T easy vector (Promega) and 10 clones/each cell line were sequenced by using T7/Sp6 primers (Eurofins MWG Operon).

Real-time PCR. To quantify p65BTK mRNA immunoprecipitated in RIP experiments cDNA was synthesized using a High Capacity RNA to cDNA Master Mix and analysed by quantitative PCR (qPCR) using customized TaqMan gene expression assays on a 7900HT Real-Time PCR system (all Applied Biosystems). Amplification specific for p65BTK isoform was performed using the following primers:

p65FW: 5' CCCATTATGTGGCAGGCACT 3'

p65REV: 5'CTTCAGAAAGATGCTCTCCAGA 3'

p65PROBE: 5' TGAACTCCAGAAAGAAGAA 3'

p65BTK gene expression was normalized to phosphoglycerate kinase expression (#Hs99999906_m1, Applied Biosystem), and expressed as fold-change of control samples.

SUPPLEMENTARY FIGURES

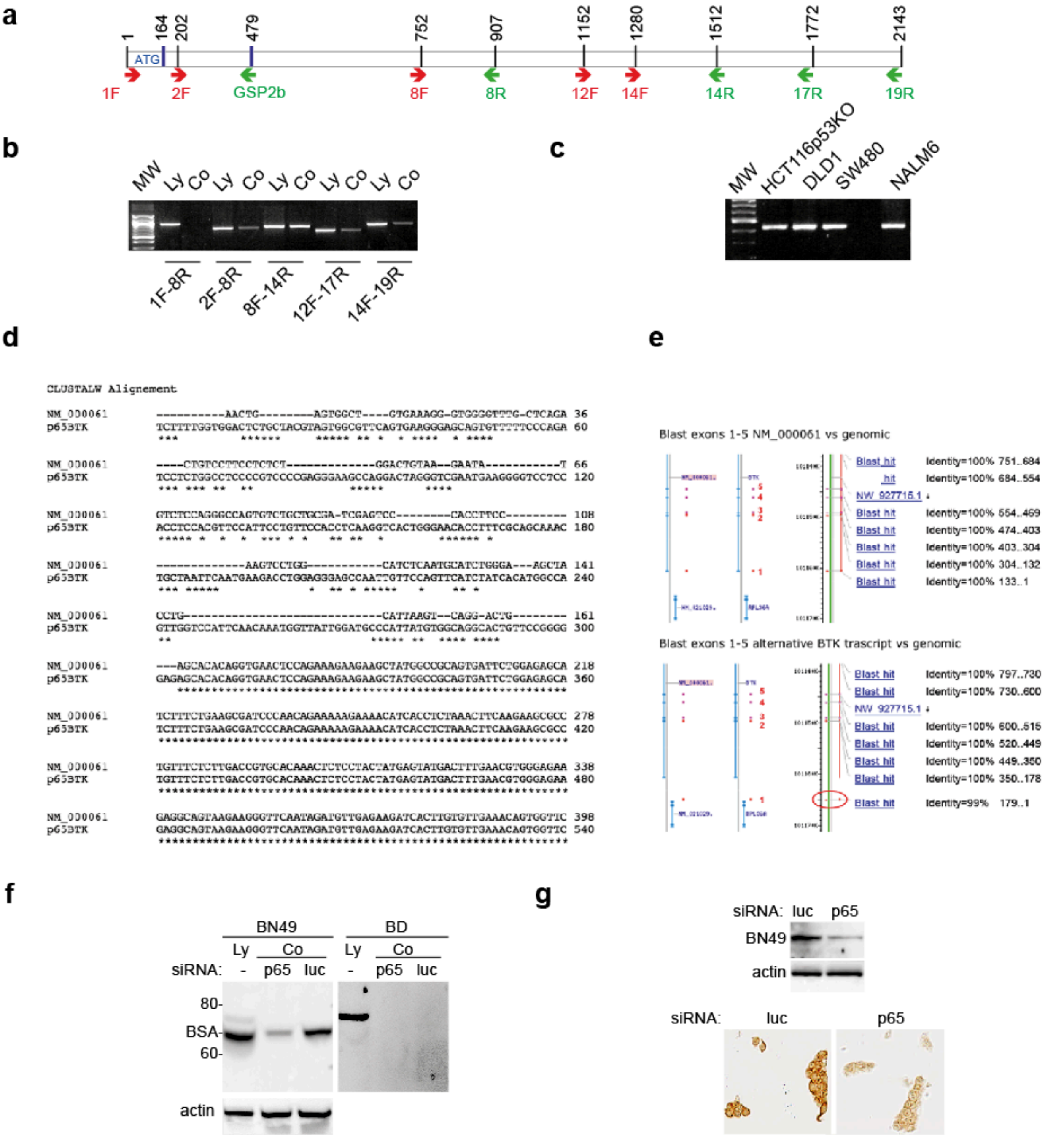

Supplementary Figure 1. Cloning p65BTK and anti-p65BTK antibodies characterization. (**a**) Scheme of the primers used to amplify BTK from RNA of colon cancer and lymphoid cell lines and to perform RACE experiments. Forward (F) and reverse (R) primers were labelled with numbers corresponding to the BTK exon sequence (NM_000061) where the primer anneals. GSP2b primer was used for 5'RACE. (**b**) PCR was performed using primers annealing on different exons of BTK mRNA (NM_000061) and cDNAs reverse transcribed from RNA extracted from lymphoid (Ly) and colon (Co)

cancer cells. (**c**) cDNA retro-transcribed from RNAs extracted from colon and lymphoid cancer cells, was PCR amplified using a forward primer annealing in exon 1b and a reverse primer annealing on the second common exon. (**d**) colon cancer-derived mRNA contains a first exon (exon 1b) different from the one expressed in B cells (exon 1a). ClustalW alignment of p77BTK (NM_000061) with p65BTK sequence identified by 5'RACE PCR in colon cancer cell lines. Only the alignment of the exon 1 and the first 50 nucleotides of exon 2 are shown. (**e**) BLAST alignment of the first 5 exons from p77BTK cDNA (NM_000061) and p65BTK cDNA vs. genomic DNA. (**f**) Characterization of BN49 polyclonal antibodies. *Left*: Western blot analysis of lysates from colon cancer cells (Co: HCT116) transfected with control (luc) or p65BTK-specific siRNA and harvested after 48hs. Lysates from lymphoid leukemia cells (Ly: Nalm6) were used as controls expressing p77BTK. Bovine serum albumin (BSA, MW 66 kDa) was used as MW marker. Bound antibodies were revealed using a chemiluminescent system. *Right*: The same blot was re-incubated with a monoclonal antibody raised against the PH domain-containing N-term of p77BTK (# 611117 BD Transduction laboratories) and therefore not reacting with p65BTK: immunoreactivity was revealed using a fluorescent secondary antibody (AlexaFluor488, Molecular Probes). (**g**) Top: Western blot analysis of lysates from SW480 colon cancer cells harvested 48hs after transfection with control (luc) or p65BTK-specific siRNA and used to produce cells blocks. Bottom: IHC using BN49 on slides from cells blocks.

a UCCC putative hnRNPK binding sites

UCAACAAAUG upstream Open Reading Frames (uORFs)

AUG start of translation in p77BTK (*GCU**AUG**GCC* italic underlined letters surrounding the starting codon indicate the Kozak consensus sequence)

AUG start of translation in p65BTK

p65 whole 5'UTR

UCUUUUGCUGGACUCUGCCUACCUACUGCCCUUCACUGAAGCGACCAGUGUUUUUCCCAGAUCCUCUGCCCUCCCCCGUCCCCCGACCG
AAGCCAGGACUAGG*GUCGAAUGAAGGGGUCCUCCACCUCCACGUUCCAUUCCUGUUCCACCUCAAGGUCACUGGGAACACCUUUCG*
*CAGCAAACUGCUAA*UUCA*AUGAAGACCUGGAGGGAGCCAAUUGUUCCAGUUCAUCUAUCACAUGGCCAGUUGGUCCAUUCAACAAA*
*UGGUUAUUGGAUGCCCAUUAUGUGGCAGGCACUGUUCCGGGGGAGAGCACACAGGUGA*ACUCCAGAAAGAAGAA*GCU**AUG**GCC*GCA
GUGAUUCUGGAGAGCAUCUUUCUGAAGCGAUCCCAACAGAAAAAGAAAACAUCACCUCUAAACUUCAAGAAGCGCCUGUUUCUCUU
GACCGUGCACAAACUCUCCUACUAUGAGUAUGACUUUGAACGUGGGAGAAGAGGCAGUAAGAAGGGUUCAAUAGAUGUUGAGAAGA
UCACUUGUGUUGAAACAGUGGUUCCUGAAAAAAAUCCUCCUCCAGAAAGACAGAUUCCGAGAAGAGGUGAAGAGUCCAGUGAA***AUG***

b

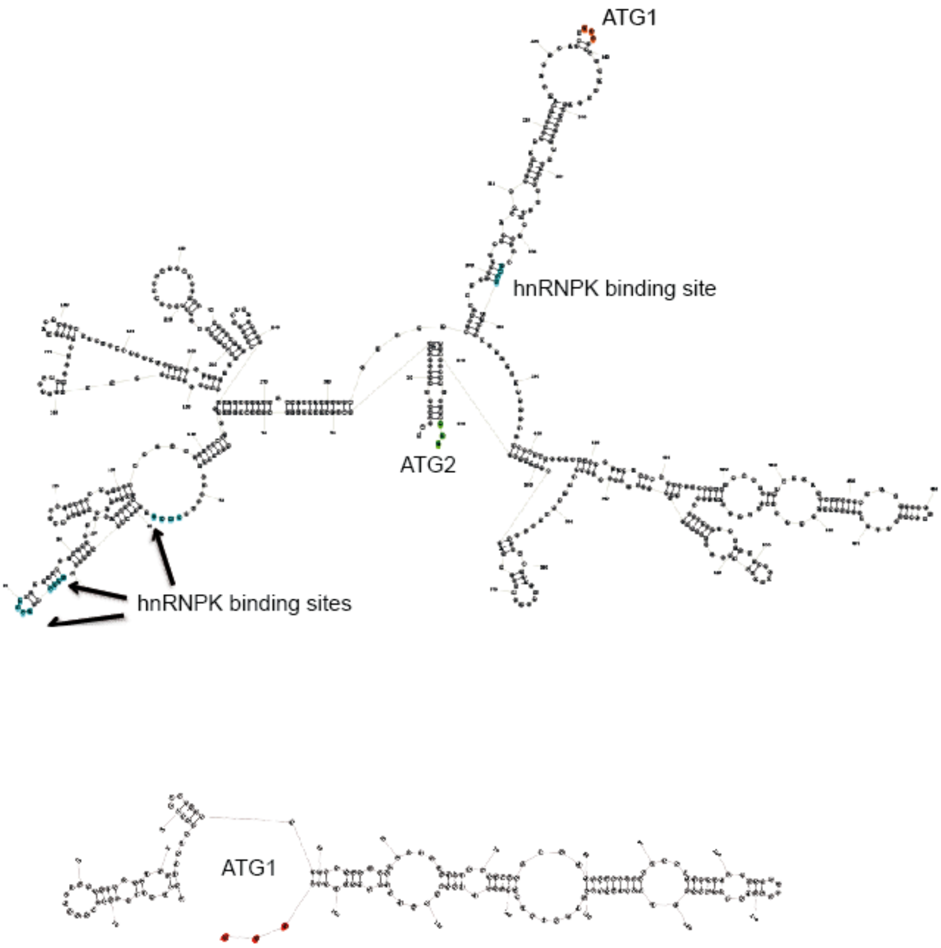

Supplementary Figure 2. hnRNPK sites in p65BTK-encoding mRNA. (**a**) Bioinformatics' analysis of the 5'UTR of p65BTK messenger showing four putative hnRNPK binding sites. (**b**) 5'UTR sequences from p65BTK-encoding and p77BTK-encoding mRNAs were analysed and compared using AveRNA application from the RNAsoft package (http://www.rnasoft.ca) and the structures visualized by the VARNAv3-7.jar application (fr.orsay.lri.varna.applications.VARNAGUI). ATG1, ATG2 and hnRNPK binding sites are indicated. *Top*: 5'UTR structure of the mRNA encoding p65BTK. *Bottom*: 5'UTR structure of the mRNA encoding p77BTK.

a

http://iresite.org/

Sequences producing significant alignments:	Score (bits)	E Value
IRESite_Id:110 Apaf-1 gene	29	3.1
IRESite_Id:223 UtrA gene	28	10
IRESite_Id:140 EMCV-R virus	28	10
IRESite_Id:465 plasmid pUTRA/CAT with functional UtrA IRES from ...	28	10
IRESite_Id:238 plasmid pbetaGAL/UtrA/CAT with functional UtrA IR...	28	10
IRESite_Id:239 plasmid (deltaCMV)betaGAL/UtrA/CAT with functiona...	28	10
IRESite_Id:437 RhPV_IGR virus	26	35
IRESite_Id:437 RhPV_5NCR virus	26	35
IRESite_Id:59 PSIV_IGR virus	26	35
IRESite_Id:68 idefix virus	26	35
IRESite_Id:491 AQP4 gene	26	35

b

Sequences producing significant alignments:	Score (bits)	E Value
IRESite_Id:59 2D structure of transcript of PSIV_IGR virus	23	1.2
IRESite_Id:140 2D structure of transcript of EMCV-R virus	21	3.9
IRESite_Id:35 2D structure of transcript of c-myc gene	21	3.9
IRESite_Id:597 2D structure of transcript of TMEV virus	19	13
IRESite_Id:222 2D structure of transcript of HCV_type_1a virus	19	13
IRESite_Id:222 2D structure of transcript of HCV_type_1a virus	19	13
IRESite_Id:222 2D structure of transcript of HCV_type_1a virus	19	13
IRESite_Id:42 2D structure of transcript of HAV_HM175 virus	19	13
IRESite_Id:42 2D structure of transcript of HAV_HM175 virus	19	13
IRESite_Id:519 2D structure of transcript of FGF1A gene	19	13
IRESite_Id:225 2D structure of transcript of CVB3 virus	19	13
IRESite_Id:491 2D structure of transcript of AQP4 gene	19	13
IRESite_Id:359 2D structure of transcript of plasmid pXLCSFV1-44...	19	13
IRESite_Id:482 2D structure of transcript of plasmid pRKMI2F wit...	19	13
IRESite_Id:481 2D structure of transcript of plasmid pRKMI1F wit...	19	13
IRESite_Id:541 2D structure of transcript of plasmid pBiCAQP4 wi...	19	13
IRESite_Id:109 2D structure of transcript of XIAP_305-466 gene	17	44
IRESite_Id:109 2D structure of transcript of XIAP_5-464 gene	17	44
IRESite_Id:471 2D structure of transcript of MYB gene	17	44
IRESite_Id:486 2D structure of transcript of plasmid pbetaGAL/5'...	17	44

c

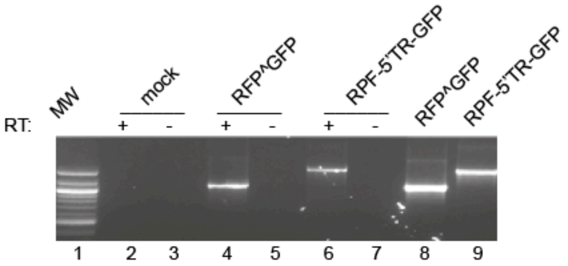

Supplementary Figure 3. 5'UTR of p65BTK mRNA contain an IRES but not a cryptic promoter. (**a**, **b**) The putative IRES sequence in the 5'UTR of p65BTK mRNA was identified by searching against the IRESite database (http://www.iresite.org) for sequences producing significant alignments. List of sequences producing significant alignment as (**a**) a linear sequence and (**b**) a secondary structure**.** (**c**) The presence of a cryptic promoter was ruled out by verifying that a unique messenger coding for both RFP and GFP is transcribed in transfected cells. cDNA from HeLa cells upon mock transfection (mock) or transfection with a bi-cistronic vector encoding CMV-regulated RFP and promoterless-GFP (RFP^GFP) or GFP under the control of p65BTK 5'UTR (RFP-5'UTR-GFP) was amplified using a forward primer annealing in the RFP sequence and a reverse primer annealing in the GFP sequence. Products of the retro-transcription reaction in absence (RT -: lanes 3, 5, 7) or presence of reverse transcriptase (RT +: lanes 2, 4, 6) followed by PCR amplification were visualized in 1% agarose gel. PCR products, amplified using RFP^GFP

and RFP-5'UTR-GFP plasmids as templates, loaded in lane 8 and 9, respectively, showed the same size of the products obtained using as templates the cDNA from the cells transfected with the correspondent plasmids.

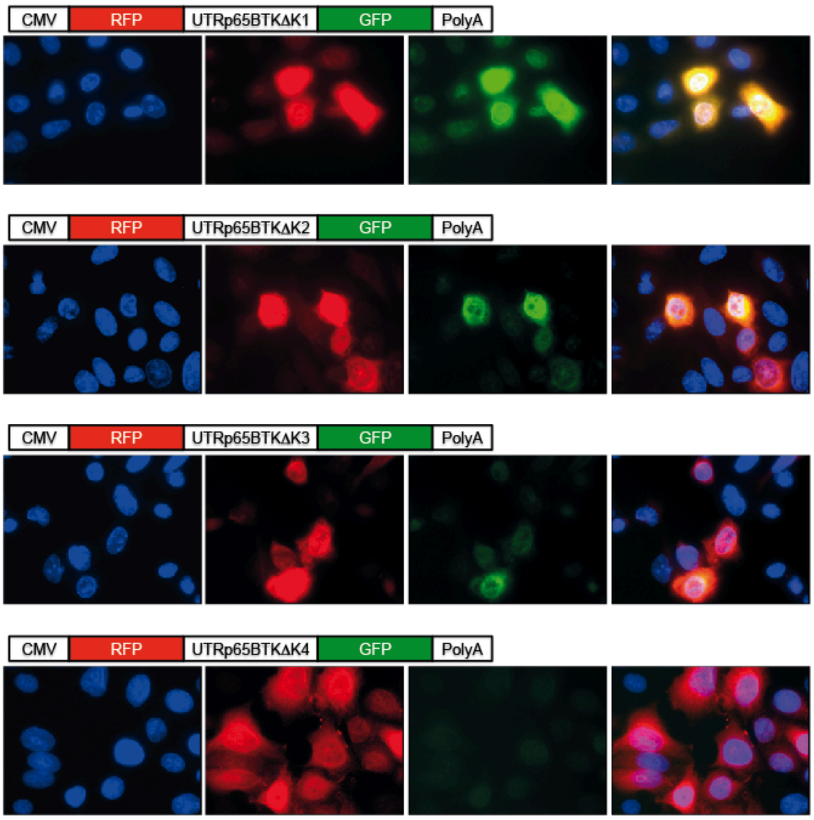

Supplementary Figure 4. IRES-dependent translation of p65BTK depends on hnRNPK. Fluorescence of HeLa cells upon transfection with progressive deletion mutants lacking: the first hnRNPK binding sites present in the 5'UTR of p65BTK mRNA (ΔK1) (first row); the first and the second hnRNPK binding sites present in the 5'UTR of p65BTK mRNA (ΔK2) (second row); the first, the second and the third hnRNPK binding sites present in the 5'UTR of p65BTK mRNA (ΔK3) (third row); all four (ΔK4) hnRNPK binding sites present in the 5'UTR of p65BTK mRNA (fourth row). DAPI was used to stain nuclei.

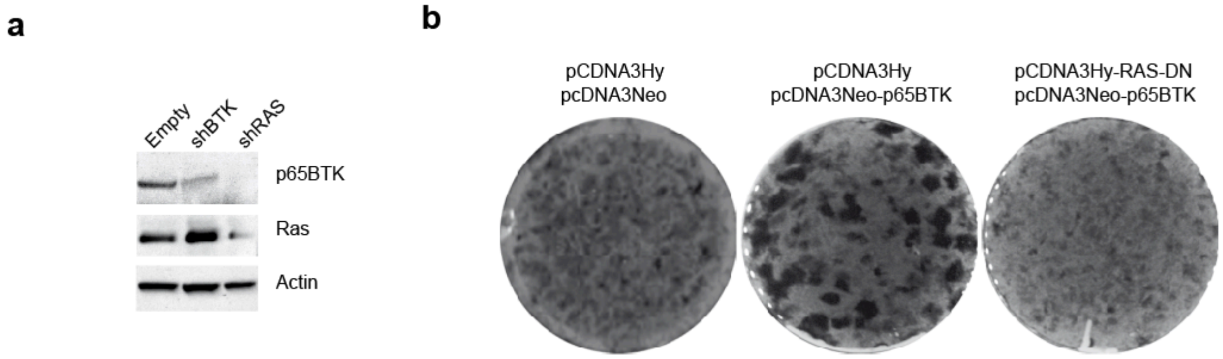

Supplementary Figure 5. p65BTK and RAS expression and transforming activities. (**a**) NIH3T3 cells transfected with empty vector or plasmids encoding shBTK, and shRAS, tested 48 after transfection. (**b**) Focus assay of NIH3T3 cells stably transfected with empty vectors and RASV-DN, and then transiently transfected with p65BTK expression plasmid. Plates were stained with crystal violet 2 weeks after transfection.

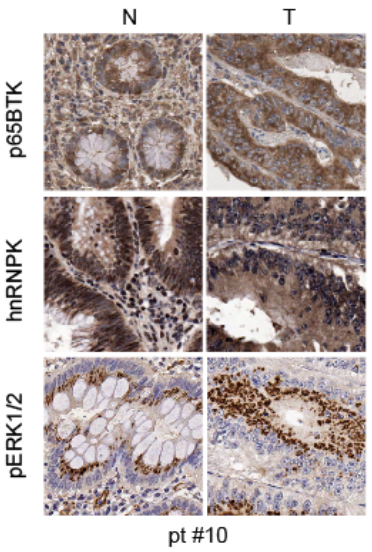

Supplementary Figure 6. p65BTK expression, cytoplasmic hnRNPK accumulation and ERKs activation. Immunohistochemical detection of p65BTK, hnRNPK and pERK1/2 in formalin-fixed paraffin-embedded specimens. p65BTK staining was performed by using BN49 polyclonal antibody, counterstaining with Haematoxylin and Eosin. 40x magnification. A single peritumoural sample with very high p65BTK expression shows also intense cytoplasmic hnRNPK staining and high pERK1/2 activation.

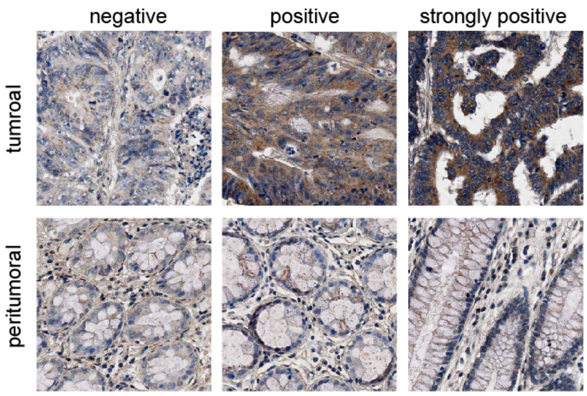

Supplementary Figure 7. p65BTK expression in colon cancers. Examples of p65BTK staining, graded accordingly to an increasing intensity by blind reading by 2 experienced operators**.** p65BTK staining was performed by using BN49 polyclonal antibody, counterstaining with Haematoxylin and Eosin. 40x magnification.

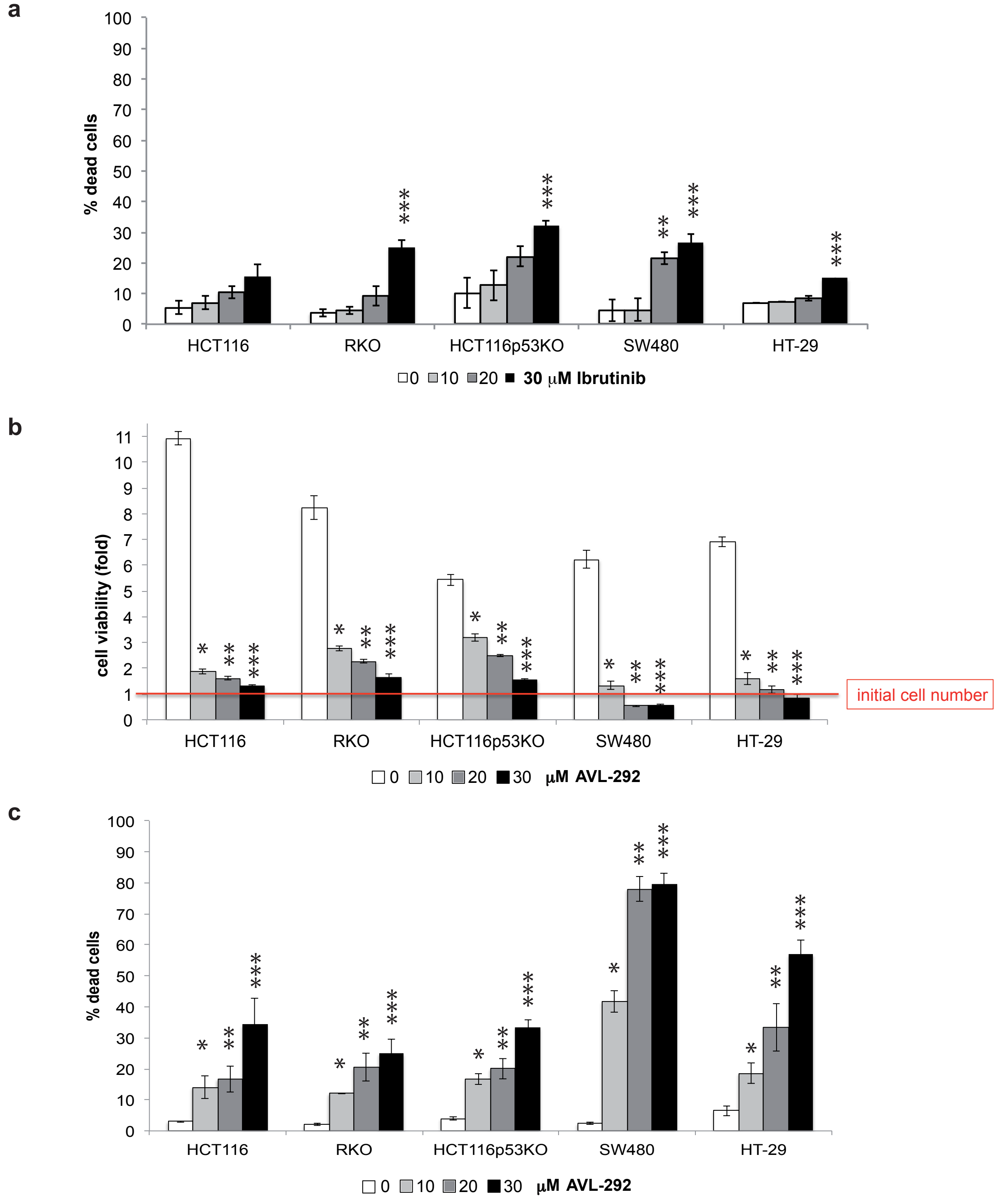

Supplementary Figure 8. BTK inhibitor AVL-292 exherts a stronger effect on growth and survival of colon cancer cells than Ibrutinib. (**a**) Cell death was assessed after 72 hs treatment with the indicated concentrations of Ibrutinib by Trypan blue staining. Data are the average from 3 independent experiments; error bars show SEM. *** 30 μM vs 0 μM Ibru: $p< 0.05$; ** 20 μM vs 0 μM Ibru: $p< 0.05$. (**b**) Cell viability was assessed after 72 hs treatment with the indicated concentration of AVL-292; crystal violet assay was performed to quantify viable cells; data is presented as fold change of the initial cell number obtained from three independent experiments; error bars show SEM. * 10 μM vs 0 μM Ibru: $p< 0.05$; ** 20 μM vs 0 μM Ibru: $p< 0.05$; *** 30 μM vs 0 μM Ibru: $p< 0.05$. (**c**) Cell

death was assessed after 72 hs treatment with the indicated concentrations of AVL-292 by Trypan blue staining. Data are the average from 3 independent experiments; error bars show SEM. * 10 µM vs 0 µM Ibru: $p < 0.05$; ** 20 µM vs 0 µM Ibru: $p < 0.05$; *** 30 µM vs 0 µM Ibru: $p < 0.05$.
