# Supplementary material for: A novel oncogenic BTK isoform is overexpressed in colon cancers and required for RAS-mediated transformation
Source: Oncogene. 2016 Jan 25;35(33):4368–78. doi: 10.1038/onc.2015.504 (PMC4994017; doi:10.1038/onc.2015.504)
Supplement: Supplementary file 2 — Supplementary Table 1 (PDF 46 kb) [file 41388_2016_BFonc2015504_MOESM55_ESM.pdf]

Patients from Desio Hospital							
	sex	age	diagnosis	grade	stage	lymph nodes	metastasis
#1	F	59	villous adenoma	0	in situ	0	0
#2	M	73	adenoca	3	III	3	0
#3	M	80	adenoca	2	II	0	0
#4	M	78	adenoca	2	II	1	0
#5	M	82	adenoca	1	II	0	0
#6	M	59	adenoca	3	III	0	liver, peritoneum
#7	M	78	adenoca	2	II	0	0
#8	M	67	adenoca	2	III	1	liver
#9	M	70	adenoca	3	II	0	0
#10	F	90	adenoca	1	II	0	0
#11	M	74	adenoca	2	IV	1	liver
#12	F	51	adenoca	2	II	1	0
#13	M	55	adenoca	3	III	1	0

Patients from Trieste cohort							
	sex	age	diagnosis	grade	stage	lymph nodes	metastasis
#1	M	72	adenoca	1	II	0	0
#3	F	59	adenoca	2	II	0	0
#5	M	72	adenoca	2	II	0	0
#7	M	63	adenoca	2	II	0	0
#9	M	64	adenoca	2	II	0	0
#11	M	70	adenoca	1	II	0	0
#13	F	63	adenoca	2	II	0	0
#15	F	69	adenoca	1	II	0	0
#17	M	69	adenoca	2	II	0	0
#19	M	68	adenoca	2	II	0	0
#21	F	56	adenoca	2	II	0	0
#23	F	46	adenoca	2	II	0	0
#25	F	77	adenoca	2	II	0	0
#27	F	59	adenoca	2	II	0	0
#29	M	60	adenoca	2	II	0	0
#31	F	67	adenoca	2	II	0	0
#33	M	61	adenoca	2	II	0	0
#35	F	67	adenoca	2	II	0	0
#37	M	73	adenoca	2	II	0	0
#39	M	81	adenoca	2	II	0	0
#41	M	66	adenoca	2	II	0	0
#43	M	76	adenoca	2	II	0	0
#45	F	76	adenoca	1	II	0	0
#47	F	61	adenoca	2	II	0	0
#49	F	68	adenoca	2	II	0	0
#51	F	72	adenoca	1	II	0	0
#53	F	80	adenoca	2	II	0	0
#55	M	84	adenoca	2	II	0	0
#57	F	71	adenoca	2	II	0	0
#59	M	82	adenoca	2	II	0	0
#61	M	72	adenoca	2	II	0	0
#63	F	59	adenoca	2	II	0	0
#65	M	54	adenoca	2	II	0	0
#67	F	66	adenoca	1	II	0	0
#69	M	59	adenoca	2	II	0	0
#71	M	65	adenoca	2	II	0	0
#73	M	64	adenoca	2	II	0	0
#75	M	69	adenoca	2	II	0	0
#77	M	79	adenoca	2	II	0	0
#79	M	58	adenoca	2	II	0	0
#81	F	56	adenoca	2	II	0	0
#83	M	58	adenoca	2	II	0	0

Supplementary Table 1. Patients characterization. In the table are shown: age, sex, diagnosis, TNM classification and grade.
